# Immune regulation and emerging roles of noncoding RNAs in *Mycobacterium tuberculosis* infection

**DOI:** 10.3389/fimmu.2022.987018

**Published:** 2022-10-13

**Authors:** Shufan Liang, Jiechao Ma, Hanlin Gong, Jun Shao, Jingwei Li, Yuejuan Zhan, Zhoufeng Wang, Chengdi Wang, Weimin Li

**Affiliations:** ^1^ Department of Respiratory and Critical Care Medicine, Med-X Center for Manufacturing, Frontiers Science Center for Disease-Related Molecular Network, West China Hospital, West China School of Medicine, Sichuan University, Chengdu, China; ^2^ Artificial Intelligence (AI) Lab, Deepwise Healthcare, Beijing, China; ^3^ Department of Integrated Traditional Chinese and Western Medicine, West China Hospital, Sichuan University, Chengdu, China

**Keywords:** *Mycobacterium tuberculosis*, microRNA, long noncoding RNA, circular RNA, immune regulation, biomarker

## Abstract

Tuberculosis, caused by *Mycobacterium tuberculosis*, engenders an onerous burden on public hygiene. Congenital and adaptive immunity in the human body act as robust defenses against the pathogens. However, in coevolution with humans, this microbe has gained multiple lines of mechanisms to circumvent the immune response to sustain its intracellular persistence and long-term survival inside a host. Moreover, emerging evidence has revealed that this stealthy bacterium can alter the expression of demic noncoding RNAs (ncRNAs), leading to dysregulated biological processes subsequently, which may be the rationale behind the pathogenesis of tuberculosis. Meanwhile, the differential accumulation in clinical samples endows them with the capacity to be indicators in the time of tuberculosis suffering. In this article, we reviewed the nearest insights into the impact of ncRNAs during *Mycobacterium tuberculosis* infection as realized *via* immune response modulation and their potential as biomarkers for the diagnosis, drug resistance identification, treatment evaluation, and adverse drug reaction prediction of tuberculosis, aiming to inspire novel and precise therapy development to combat this pathogen in the future.

## Introduction

Tuberculosis (TB), caused by *Mycobacterium tuberculosis* (*M. tuberculosis*), poses a severe threat to public health, with approximately 10 million new cases and 1.5 million deaths reported in 2020 according to the World Health Organization (WHO). Much effort has been devoted to preventing the spread of the contagious disease. Nevertheless, the emergence of coronavirus disease 2019 (COVID-19) reversed the death toll of TB back to the level equivalent to that in 2017 (1). In addition, the proportion of drug-resistant tuberculosis (DR-TB) has steadily increased, resulting in an exacerbated challenge to TB supervision (2). Thus, precise indicators for the diagnosis and remedy direction of TB are urgently needed. The exploitation of novel biomarkers and host-directed therapy (HDT) may provide opportunities to surmount these conundrums.

*M. tuberculosis*, an intracellular pathogen, has evolved ingenious strategies to evade host immune defenses, armed with a tenacious power to proliferate in innate immune cells (3). Macrophages constitute the predominant niche of resident *M. tuberculosis* while functioning as the first line of self-defense, inducing innate immune cell responses such as cytokine secretion, autophagy, and apoptosis, and participating in acquired immunological reactions to eradicate this cunning microbial enemy (4).

Up to 90% of the human genome is transcribed into ribose nucleic acids (RNAs). However, only 1.5%–2.0% of them possess the ability to manufacture particular protein production (5). Recently, accumulating evidence has led to a reevaluation of the perception that the nonprotein-coding section of the genome is merely spurious transcriptional noise. In fact, some sequences in this proverbial “dark matter” can encode specific functional noncoding RNAs (ncRNAs) that play pivotal roles in biological regulation including immunology (6–9). Advances in RNA sequencing have led to the discovery of a multitude of ncRNAs including microRNAs (miRNAs), long noncoding RNAs (lncRNAs), circular RNAs (circRNAs), and PIWI-interacting RNAs (piRNAs) (10–12). Currently, the roles played by ncRNAs in eukaryotic cellular process mediation, ranging from gene regulation on a molecular scale to macroscopic manipulation of disease-inducing mechanisms, have been illuminated (13, 14). For instance, ncRNAs significantly influence bacterial and viral infections (15, 16). Specifically, in immunology, numerous studies have resolved the mystique of ncRNAs, which are implicated in multifarious events such as immune cell development and immune escape (17, 18). Furthermore, the contribution of ncRNAs to the underlying pathogenesis of *M. tuberculosis* in hosts has also been enumerated (19). The significant discovery of the host-pathogen interactions has ushered in an inspiring time for HDT exploration (20). Moreover, previous effort has revealed the feasibility of using these seemingly inconspicuous molecules as biomarkers of TB (21, 22).

**Figure 1:**
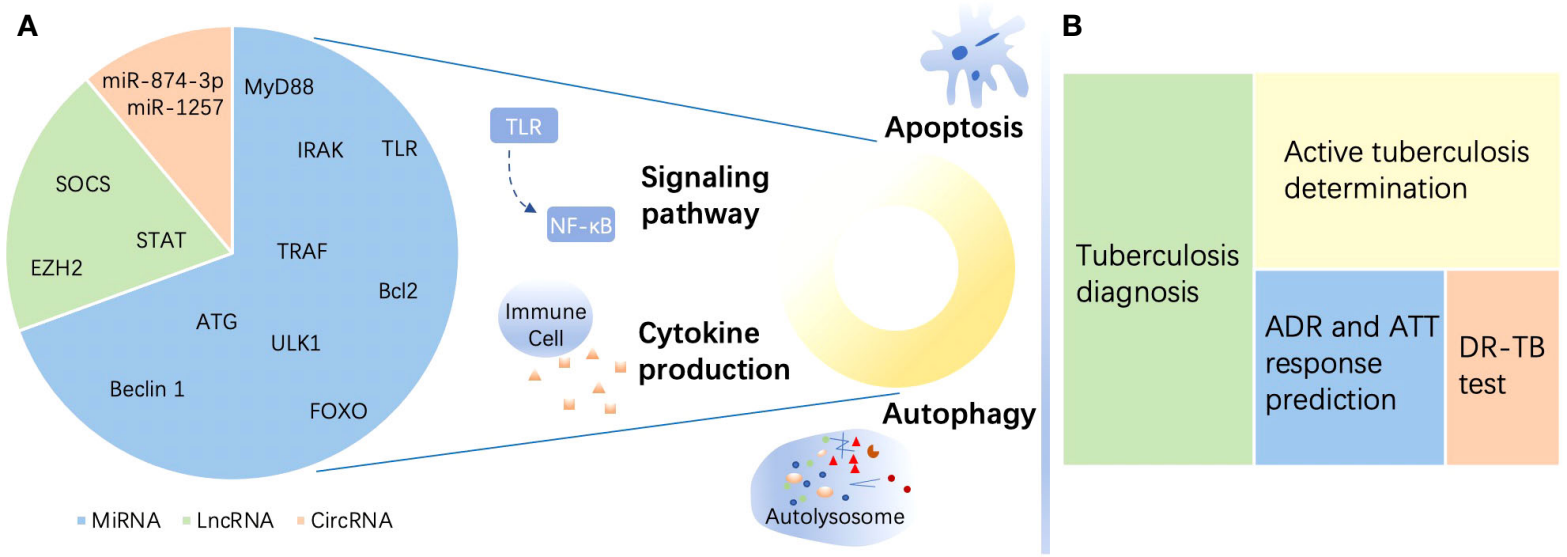
A brief summary of the roles of noncoding RNAs in tuberculosis. **(A)** Targets of noncoding RNAs (ncRNAs) in immune regulation in tuberculosis (TB) and estimated proportion of studies directed to the three ncRNAs enumerated in this review. MicroRNAs (miRNAs) mostly target myeloid differentiation factor 88 (MyD88), Toll-like receptor (TLR), tumor necrosis factor (TNF) receptor-associated factor (TRAF), and interleukin-1R-associated kinase (IRAK) to regulate signaling pathways and target Unc-51-like autophagy-activating kinase 1 (ULK1), autophagy-related gene (ATG), and beclin 1 to affect autophagy. Moreover, miRNAs target the Bcl-2 family and Forkhead box transcription factor class O (FOXO) to modulate apoptosis during TB. Long noncoding RNAs (lncRNAs) regulate the immune response in the host by targeting enhancer of zeste homolog (EZH) 2, suppressor of cytokine signaling (SOCS), signal transducer and activator of transcription (STAT), etc. Circular RNAs (circRNAs) perform immunity functions by acting as miRNA sponges. **(B)** ncRNAs serve as biomarkers in TB. ncRNAs show the potential to be biomarkers of different functions including diagnosis, active individual determination, adverse drug reaction (ADR) and response to antituberculosis therapy (ATT) monitoring, and drug resistance prediction of TB. DR-TB, drug-resistant tuberculosis.

In this review, we elaborated on the promising roles of ncRNAs, focusing on miRNAs, lncRNAs, and circRNAs, in TB. We described the regulatory impact of the three ncRNAs on immune function in disparate parts. In each section, we offered a brief introduction to the foundational structure and function of the highlighted ncRNAs, followed by a concrete explanation of their effects on *M. tuberculosis* infection. Then, we narrated their potential as biomarkers for TB diagnosis, DR-TB identification, and treatment monitoring. Finally, we summarized the included studies with achievements and existing challenges for the purpose of utilizing ncRNAs for TB management, including rapid diagnosis and precise treatment. The major content of the study is illustrated succinctly in **Figure 1**.

## Immune regulation of miRNAs in tuberculosis

### A concise description of miRNAs

miRNAs, approximately 22 nucleotides in length, are endogenous single-stranded small ncRNAs with highly conserved structure, emerging as posttranscriptional epigenetic modulators after binding to the 3′ untranslated regions (3′ UTRs) of targeting messenger RNAs (mRNAs), resulting in mRNA decay or destabilization (23–25). Numerous studies have authenticated the polytropic roles of miRNAs in multiple biological processes, from cell differentiation to disease occurrence (26, 27). In addition, miRNAs exert a crucial influence on immune response regulation (28, 29). When certain morbific microorganisms stealthily enter the human body, miRNAs will respond to modulate the immune response in various ways (30). Focused on *M. tuberculosis*, previous studies have revealed that this crafty pathogen can alter the expression of host miRNAs, enabling this microorganism to evade immune clearance and achieve long-term dormancy inside the body (31). In innate immune reactions, macrophages serve as the first-line defense in the face of the complex enemy microorganism invasion (32), and the aftermath of the war between *M. tuberculosis* and macrophages determines the infection outcome-latent or active TB. Moreover, *M. tuberculosis* has developed various strategies to subvert sthe host immune response, such as autophagy (33). Hence, we enumerated the miRNA modulation caused by *M. tuberculosis* and the subsequent influence of this regulatory effect on antimicrobial immunity.

### miRNA-regulated signaling pathways in *M. tuberculosis* infection

**Figure 2:**
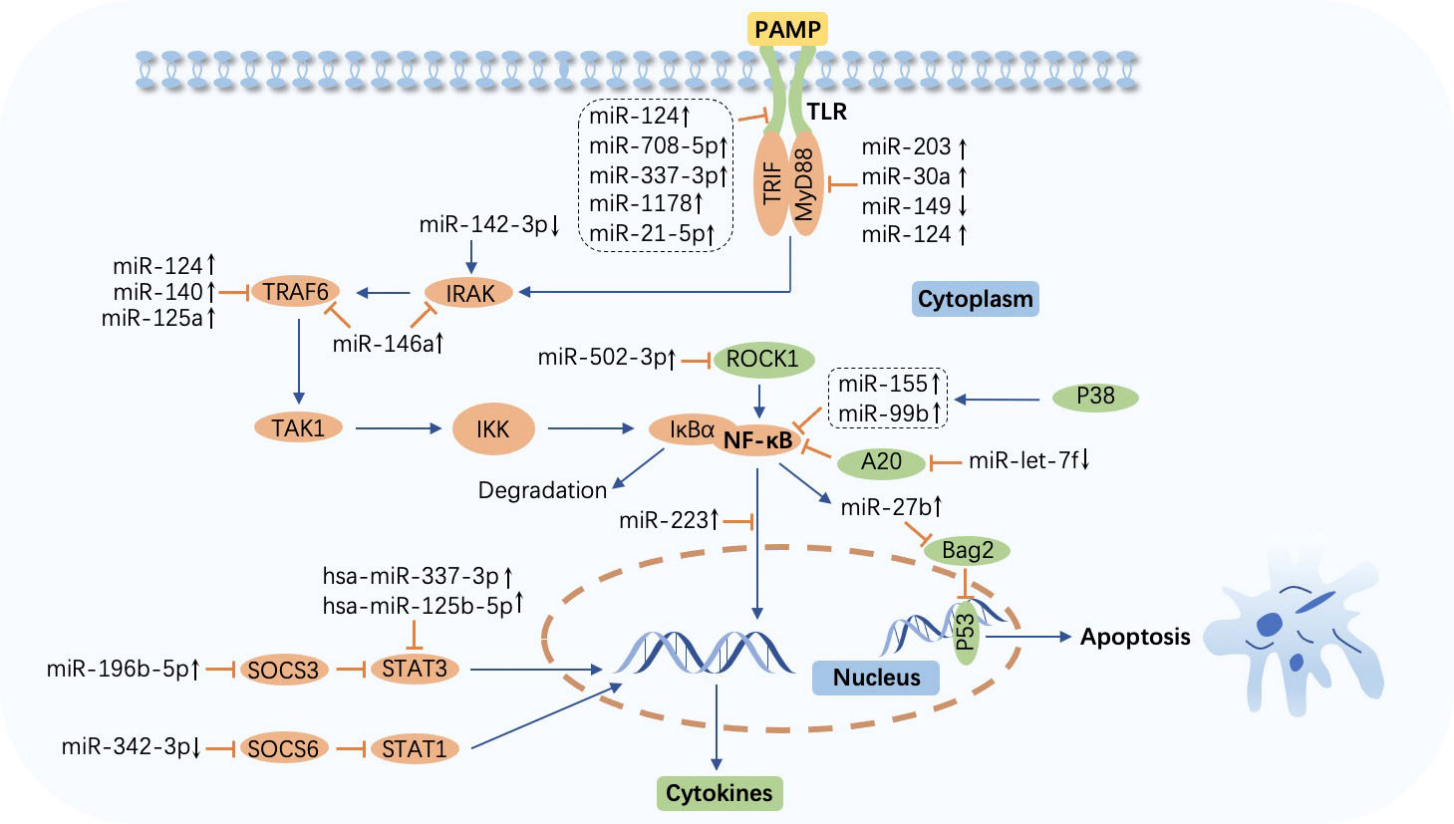
MicroRNA-regulated signaling pathway during *Mycobacterium tuberculosis* infection. The Toll-like receptor (TLR)/nuclear factor-κB (NF-κB) pathway is activated by the combination of TLRs and pathogen-associated molecular patterns (PAMPs) and impacts cytokine production. Abundant molecules such as TAK1 and IκB kinase (IKK) participate in this process. IκBα is degraded after the release of NF-κB from IKK. Overexpressed or downregulated microRNAs (miRNAs) target myeloid differentiation factor 88 (MyD88), Toll-interleukin (IL)-1-resistance (TIR) domain-containing adaptor inducing interferon-β (TRIF), IL-1R-associated kinase (IRAK), tumor necrosis factor (TNF) receptor-associated factor (TRAF), NF-κB, suppressor of cytokine signaling (SOCS), signal transducer and activator of transcription (STAT), and p53 to affect host immune cell response in tuberculosis. miR-27b is induced by NF-κB and triggers p53-dependent apoptosis by targeting Bcl-2 associated athanogene 2 (Bag2). ↑, upregulated; ↓, downregulated; ⟶, stimulate; ⟞, inhibit.

Toll-like receptors (TLRs), a family of pattern recognition receptors (PRRs) that reside on the plasma membrane of macrophages and other tissues involved in immunity, sense pathogen-associated molecular patterns (PAMPs) such as lipopolysaccharide (LPS) in Gram-negative bacteria and single-stranded RNAs. Subsequently, adaptor proteins including myeloid differentiation factor 88 (MyD88), Toll-interleukin (IL)-1-resistance (TIR) domain-containing adaptor-inducing interferon-β (TRIF), TRIF‐related adaptor molecule (TRAM), and TIR‐containing adaptor protein (TIRAP) (34–36), are engaged by TIRs, cytoplasmic domains of TLRs, after which IL-1R-associated kinase (IRAK), tumor necrosis factor (TNF) receptor-associated factor (TRAF), and IκB kinase (IKK) complex are recruited by appropriate signals in turn. The activation of IKK leads to the phosphorylation and degradation of IκB in the canonical nuclear factor-κB (NF-κB) pathway, releasing NF-κB, a two-subunit nuclear transcription factor that dimerizes and is further activated through different posttranscriptional modifications. NF-κB is then translocated into the nucleus to regulate corresponding gene expression by binding to specific DNA sequences, ultimately eliciting inflammatory cytokine secretion to eradicate invading pathogens (37–40). The role of miRNAs in modulating signaling pathway cascades, including the TLR/NF-κB pathway during *M. tuberculosis* infection, which results in the suppression or enhancement of the immune response, has been explored as follows (**Figure 2**; **Table 1**).

**Table 1:** MicroRNA-regulated signaling pathways in tuberculosis.

miRNAs	Expression	Targets	Biological function	Ref.
miR-203	↑	MyD88	Suppresses NF-κB signaling and production of TNF-α and IL-6	(41)
miR-149	↓	MyD88	Promotes the production of NF-κB1, TNF-α, and IL-6	(42)
miR-30a	↑	MyD88	Suppresses the production of TNF-α, IL-6, and IL-8	(43)
miR-124	↑	MyD88, TLR6, TRAF6	Suppresses the expression of p65 NF-κB, TNF-α, and IL-6	(44)
miR-146a	↑	IRAK1, TRAF6	Suppresses the production of iNOS, NO, TNF-α, IL-1β, and IL-6	(45, 46)
miR-27a	↑	TICAM1	Inhibits TLR3-related immune response	(47)
miR-142-3p	↓	IRAK1	Promotes the production of NF-κB1, TNF-α, and IL-6	(48)
miR-1178	↑	TLR4	Suppresses the production of IFN-γ, IL-1β, IL-6, and TNF-α	(49)
miR-708-5p	↑	TLR4	Suppresses the production of IFN-γ, IL-1β, IL-6, and TNF-α	(50)
miR-337-3p	↑	TLR4, STAT3	Depresses vitamin D receptor-mediated antituberculosis reaction and promotes bacterial pathogenicity	(51)
hsa-miR-337-3p, hsa-miR-125b-5p	↑	STAT3	Suppress the responsiveness of Vγ2Vδ2 T cells to IL-23-induced expansion	(52)
miR-125a	↑	TRAF6	Suppresses the production of IFN-γ, IL-1β, IL-6, and TNF-α	(53)
miR-140	↑	TRAF6	Suppresses the production of IFN-γ, IL-1β, IL-6, and TNF-α	(54)
miR-27b	↑	Bag2	Suppresses the production of NF-κB, IL-1β, IL-6, TNF-α, and iNOS, and promotes the production of ROS and apoptosis	(55)
miR-let-7f	↓	A20	Suppresses the production of IL-1β, TNF-α, and NO	(56)
miR-223	↑	p65	Suppresses the production of IL-1β, IL-6, and TNF-α	(57, 58)
miR-155, miR-99b	↑	p65	Suppress the production of IL-6 and TNF-α	(59)
miR-502-3p	↑	ROCK1	Suppresses the production of IL-6, IL-1β, and TNF-α,	(60)
miR-196b-5p	↑	SOCS3	Activates STAT3 pathway and suppresses the production of IL-6 and TNF-α	(61)
miR-342-3p	↓	SOCS6	Suppresses the secretion of TNF-α, IL-1, IL-6, and CXCL15	(62)
miR-21-5p	↑	TLR4	Suppresses the production of IL-1β, IL-6, and TNF-α	(63)

miRNA, microRNA; Ref, reference; MyD88, myeloid differentiation factor 88; NF-κB, nuclear factor-κB; TNF-α, tumor necrosis factor-α; IL, interleukin; TLR, Toll-like receptor; TRAF, TNF receptor-associated factor; IRAK, IL-1R-associated kinase; iNOS, inducible nitric oxide synthase; NO, nitric oxide; IFN-γ, interferon-γ; STAT, signal transducer and activator of transcription; Bag2, Bcl-2 associated athanogene 2; ROS, reactive oxygen species; ROCK1, Rho-associated coiled-coil-forming protein kinase 1; SOCS, suppressor of cytokine signaling; CXCL, chemokine (C–X–C motif) ligand. ↑, upregulated; ↓, downregulated.

In Bacille Calmette–Guérin (BCG)-infected macrophages, miR-203 inhibits NF-κB signaling pathway activation, decreasing the expression of IL-6 and TNF-α by targeting MyD88 (41). Similarly, miR-149 and miR-30a also target MyD88 and suppress the downstream secretion of cytokines including TNF-α, IL-6, and IL-8, inhibiting pathogen clearance (42, 43). miR-124 is highly expressed *via* MyD88 activation in *M. tuberculosis*-infected alveolar macrophages (AMs), and in response, this miRNA targets MyD88, TRAF6, and TLR6 to alleviate inflammation triggered by the microorganism invasion (44). Another study demonstrated that miR-146a suppresses the inflammatory response by negatively modulating TRAF6 and IRAK1 expression in NF-κB signaling pathway cascades, repressing the generation of proinflammatory factors such as inducible nitric oxide synthase (iNOS), nitric oxide (NO), IL-6, TNF-α, and IL-1β to facilitate replication and survival of *M. tuberculosis* in macrophages (45, 46). Targeting TIR domain-containing adaptor molecule 1 (TICAM1), a TLR3 adaptor, the engagement of miR-27a stifles TLR3-related innate immune response to promote *M. tuberculosis* survival. Bone morphogenesis protein (BMP), regulated by c-Abl, is the primary molecule that induces miR-27a overexpression(47). Furthermore, a study discovered that miR-142-3p downregulates IRAK1 and suppresses the action of inflammatory mediators including TNF-α, IL-6, and NF-κB1 (a subunit of NF-κB) (48). The time- and concentrate-dependent upregulation of miR-1178 and miR-708-5p attenuates the accumulation of proinflammatory factors such as IFN-γ, TNF-α, IL-1β, and IL-6 by targeting TLR4 and subsequently enhances microorganism vitality in *M. tuberculosis*-infected macrophages (49, 50). Similarly, overexpression of miR-337-3p is implicated in enhanced *M. tuberculosis* pathogenicity and impaired vitamin D receptor (VDR)-mediated antibacterial response by depressing the TLR4 and the signal transducer and activator of transcription 3 (STAT3, a transcription activator that plays a key role in multiple biological processes such as apoptosis and immune regulation by modulating the expression of various genes) signaling pathway (51, 64, 65). Meanwhile, another study pointed out a marked increase in hsa-miR-337-3p and hsa-miR-125b-5p in patients with TB is causative of impaired STAT3 function, which results in the abrogation of IL-23-mediated expansion of Vγ2Vδ2 T cells and weakens the ability of these cells to produce anti-TB factors (52).

Overexpression of miR-125a and miR-140 inhibits NF-κB signaling and secretion of proinflammatory cytokines such as IFN-γ and IL-1β during *M. tuberculosis* infection by targeting TRAF6 directly, leading to immune response suppression and increased microbe viability. In addition, the miR-125a level is increased in a TLR4-dependent manner (53, 54). Moreover, miR-27b is induced *via* TLR2/MyD88/NF-κB cascades in *M. tuberculosis*-infected murine lungs and spleens, subsequently targeting Bcl-2-associated athanogene 2 (Bag2) and elevating p53-dependent apoptosis and reactive oxygen species (ROS) secretion rates to eliminate intracellular pathogens while forming a negative feed loop that prevents excessive inflammation induced by NF-κB (55). A20, a feedback inhibitor of the NF-κB signaling pathway, is upregulated *via* the early secreted antigenic target of 6 kDa (ESAT-6)-dependent suppression of miR-let-7f, which leads to diminished NF-κB signaling and cytokine production, resulting in pathogen maintenance in the macrophages of *M. tuberculosis*-infected mice (56). miR-223 is abundantly expressed in monocytes and monocyte-derived macrophages (MDMs) from TB patients and attenuates nuclear translocation and p65 (a subunit of NF-κB) (66) phosphorylation, resulting in suppression of NF-κB activation and inhibition of cytokine secretion, hindering *M. tuberculosis* eradication (57). Moreover, miR-223 regulates the engagement of myeloid cells by targeting chemokine (C-X-C motif) ligand (CXCL) 2, chemokine (C-C motif) ligand (CCL) 3, and IL-6, and TB infection has been shown to be exacerbated in miR-233^−/−^ animals (58). p38 is promoted by *M. tuberculosis* Rv2346c, a member of ESAT6, which induces the overexpression of miR-155 and miR-99b, leading to the inhibition of both the activation of NF-κB and secretion of cytokines including IL-6 and TNF-α, ultimately enhancing bacillary persistence and restraining the proliferation of BCG-infected macrophages (59). Targeting Rho-associated coiled-coil-forming protein kinase 1 (ROCK1), induction of miR-502-3p by *M. tuberculosis* decreases the production of TNF-α, IL-6, and IL-1β *via* the inhibition of the TLR4/NF-κB pathway, promoting pathogen survival (60).

In addition, the STAT pathway participates in inflammatory mediator production in *M. tuberculosis* infection as well. The expression of miR-196-5p is elevated in monocytes from smoking patients with TB. By repressing the suppressor of cytokine signaling (SOCS) 3 and activating the STAT3 pathway, miR-196-5p inhibits proinflammatory cytokine production and bacterial uptake (61). However, miR-342-3p facilitates the production of cytokines and chemokines such as TNF-α, IL-1, IL-6, and CXCL15 *via* SOCS6 suppression and subsequent STAT1 activation and switches the death modality from necrosis to apoptosis in *M. tuberculosis*-infected macrophages, enhancing the anti-TB immune response. Moreover, mice with higher expression of miR-342-3p are more resistant to *M. tuberculosis* (62).

The TLR/NF-κB signaling pathway is affected most frequently by miRNAs during *M. tuberculosis* infection, which results in inflammatory cytokine regulation, ultimately resulting in the prevention or facilitation of microorganism eradication. Nevertheless, studies related to the regulatory function of other signaling pathways in bacterial infection have rarely been performed, and more research is yet to be carried out. Focusing on the likely fundamental mechanisms, scientists are called to ceaselessly pursue more novel and precise miRNA-based therapies for TB in clinical practice.

### miRNA-regulated cytokine production in *M. tuberculosis* infection

Different from those focused on signaling pathways, several studies have revealed a more direct modulatory impact of miRNAs on the expression of inflammatory cytokines, as presented in brief in **Supplementary Table S1**. For instance, miR-29 is observed to inhibit interferon (IFN)-γ production to negatively control immune reactions, while in mice, competitive sponging of miR-29 leads to a higher concentration of IFN-γ and renders the mice more resistant to *M. tuberculosis* infection (67). Meanwhile, another study pointed out that in patients with active tuberculosis (ATB), upregulated miR-29 may attenuate the response of CD4^+^ T cells to *M. tuberculosis* (68). However, the relationship between the differential expression of miR-29a, a member of the miR-29 family, and IFN-γ has not yet constituted a plausible line of inquiry (69). Likewise, *M. tuberculosis* restrains the immune response by inducing miR-132 and miR-26a, which target p300, a transcriptional coactivator of IFN-γ signaling, and lead to lower IFN-γ expression and reduce responsiveness of macrophages to this lymphokine, permitting the pathogen persistence (70). miR-144* enables the reduction in TNF-α and IFN-γ secretion and T-cell proliferation, which hinders the clearance of *M. tuberculosis* (71). Lower production of TNF occurs along with overexpression of miR-125b and downregulation of miR-155 in macrophages incubated with *M. tuberculosis* lipomannan, promoting bacterial survival inside the host (72). Another study revealed that ESAT-6-induced miR-155 inhibits the expression of SH2-containing inositol 5’-phosphatase (SHIP1) and BTB and CNC homology 1 (Bach1), a repressor of haem oxygenase-1 (HO-1), and reduces IL-6 and cyclooxygenase-2 (Cox-2) production, finally facilitating *M. tuberculosis* existence in macrophages (73). Downregulating CCAAT/enhancer binding protein β (C/EBPβ), a positive transcriptional modulator of nitric oxide synthase (NOS) 2, miR-155 further decreases the synthesis of NO, thus preventing the microbe killing. When transfected with anti-miR-155, IFN-γ-activated macrophages exhibit a higher level of NO and a reduced *M. tuberculosis* burden (74).

Follistatin-like protein 1 (FSTL1) gene expression is triggered by TLR4 signaling and certain proinflammatory cytokines. In return, FSTL1 activates macrophages to promote proinflammatory cytokine and chemokine expression (75–78). In the duration of *M. tuberculosis* infection, greatly enhanced miR-32-5p targets FSTL1 and significantly attenuates the secretion of certain cytokines such as IL-1β and IL-6, which promotes inflammatory reactions, finally increasing the intracellular survival rate of *M. tuberculosis* (79). Rab10, a member of the Ras oncogene family, plays a vital role in macrophage activation. Suppressed miR-378d, which is associated with the activation of NF-κB signaling, increases the production of cytokines, including IL-1β, IL-6, and TNF-α, mediated *via* Rab10 in *M. tuberculosis*-infected macrophages and facilitates the clearance of the microbe (80). In contrast, activation of the NF-κB pathway after BCG infection induces the upregulation of miR-21, which decreases the expression of IL-12 by targeting IL-12p35 and promotes dendritic cell (DC) apoptosis by targeting Bcl2, impairing the anti-mycobacterial reactions (81). Moreover, miR-206 decreases the expression of tissue inhibitor of matrix metalloproteinase 3 (TIMP3) to elevate inflammatory cytokine secretion in THP-1 macrophages infected by *M. tuberculosis*, facilitating immune reactions against the pathogen (82). EAST6-inhibited miR-222-3p suppresses the production of proinflammatory cytokines such as IL-6, IL-1β, and TNF-α by promoting the expression of phosphatase and tensin (PTEN), which ultimately benefits microbial replication (83). In addition, BCG/H37Rv-downregulated miR-495 enables intracellular bacterial survival as a result of a superoxide dismutase 2 (SOD2)-promoted decrease in reactive oxygen species (ROS) (84).

Through various molecule targeting, miRNAs modulate the secretion of cytokines and chemokines to regulate the anti-mycobacterial response in a more straightforward manner, which gives us a hint to explore relevant therapeutic plans based on these theories.

### miRNA-regulated autophagy in *M. tuberculosis* infection

**Figure 3:**
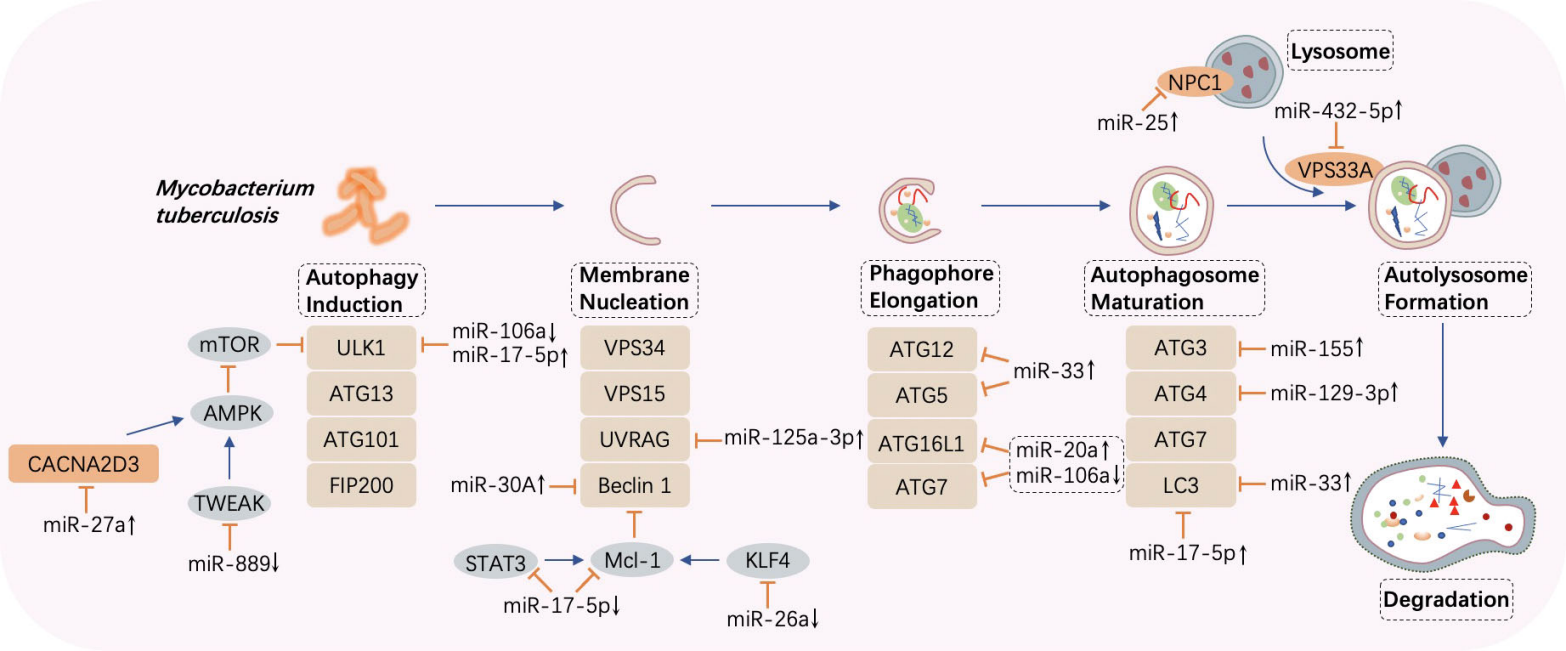
MicroRNA-regulated autophagy during *Mycobacterium tuberculosis* infection. Once autophagy is induced by *Mycobacterium tuberculosis*, several processes such as membrane nucleation to form the phagophore, elongation to realize autophagosome maturation, and the fusion between autophagosomes and lysosomes take place in turn. MicroRNAs (miRNAs) are differentially expressed and target crucial components in autophagy such as autophagy-related genes (ATGs), UV radiation resistance-associated gene (UVRAG), beclin 1, and microtubule‐associated protein 1 light chain 3 (LC3). CACNA2D3, calcium voltage-gated channel auxiliary subunit alpha2delta3; AMPK, adenosine 5’ monophosphate-activated protein kinase; TWEAK, tumor necrosis factor-like weak inducer of apoptosis; ULK1, Unc-51 like autophagy activating kinase 1; VPS, vacuolar protein sorting; STAT, signal transducer and activator of transcription; KLF, Krüpple-like factor. ↑, upregulated; ↓, downregulated; ⟶, stimulate; ⟞, inhibit.

Autophagy, an evolutionarily conserved catabolic pathway, degrades macromolecules and specific organelles through the fusion between autophagosomes and lysosomes following the entrapment of superfluous intracellular substances (85). When the bacteria invade, autophagy is mobilized to resist the pathogenic infection, playing a critical role in the innate immune response. Autophagy disruption can lead to multiple diseases (86). Moreover, research has suggested that intracellular *M. tuberculosis* elimination may be associated with autophagy (87) and that miRNAs exert a crucial impact on this cellular cargo obliteration tactic (88). Recently, mounting evidence has discovered that the contagious agent *M. tuberculosis* can combat host autophagy by interacting with autophagic components such as beclin 1, autophagy-related genes (ATGs), and microtubule‐associated protein 1 light chain 3 (LC3), which contribute significantly to essential autophagic processes including membrane nucleation and autophagosome formation and subsequent fusion with lysosomes (89). The primary miRNAs involved in the biological process and their expression and impacts are displayed in **Figure 3** and **Table 2**.

**Table 2:** MicroRNA-regulated autophagy in tuberculosis.

miRNAs	Expression	Targets	Biological function	Ref.
miR-17-5p	↓	Mcl-1, STAT3	Inhibits autophagosome formation	(90)
	↑	ULK1, LC3I/II	Inhibits autophagosome maturation	(91)
miR-20a	↑	ATG7, ATG16L	Decreases the expression of LC3 II and inhibits autophagy	(92)
miR-106a	↓	ULK1, ATG7, ATG16L1	Enhances autophagy activation	(93)
miR-129-3p	↑	Atg4b	Inhibits converting LC3I into LC3II	(94)
miR-142-3p	↑	ATG4c, ATG16L1	Inhibits phagosome maturation	(95)
miR-33	↑	ATG5, ATG12, LAMP1, LC3B, FOXO3, TFEB, AMPK	Inhibits autophagy	(96)
miR-155	↑	ATG3	Inhibits autophagosome formation and autolysosome fusion	(97)
	↑	Rheb	Enhances phagosome maturation	(98)
miR-155, miR-31	↑	Ppp2r5a	Inhibit autophagy induced by IFN-γ	(99)
miR-30A	↑	Beclin 1	Inhibits autophagy	(100)
miR-125a-3p	↑	UVRAG	Inhibits autophagosome maturation	(101)
miR-144*	↑	DRAM2	Inhibits autophagosome formation	(102)
miR-432-5p	↑	VPS33A	Inhibits autolysosome fusion	(103)
miR-542-3p	↑	VPS11	Inhibits autophagosome formation and its interplay with lysosome	(104)
miR-27a	↑	CACNA2D3	Inhibits autophagosome formation	(105)
miR-889	↑	TWEAK	Inhibits autophagosome maturation	(106)
miR-25	↑	NPC1	Impairs the function of lysosomes	(107)
miR-23a-5p	↑	TLR2	Inhibits autophagy induction	(108)
miR-26a	↓	KLF4, C/EBPβ	Inhibits autophagy and promotes M2 polarization of macrophages	(109)

miRNA, microRNA; Ref, reference; STAT, signal transducer and activator of transcription; ULK-1, Unc-51-like autophagy-activating kinase 1; LC, microtubule‐associated protein 1 light chain; ATG, autophagy-related gene; LAMP, lysosome-associated membrane protein; FOXO, Forkhead box transcription factor class O; TFEB, transcription factor EB; AMPK, adenosine 5′ monophosphate-activated protein kinase; Rheb, Ras homolog enriched in the brain; UVRAG, UV radiation resistance-associated gene; DRAM2, DNA damage regulated autophagy modulator 2; VPS, vacuolar protein sorting; CACNA2D3, calcium voltage-gated channel auxiliary subunit alpha2delta3; TWEAK, tumor necrosis factor-like weak inducer of apoptosis; TLR, Toll-like receptor; KLF, Krüpple-like factor; C/EBPβ, CCAAT/enhancer binding protein β. ↑, upregulated; ↓, downregulated.

The targets of miR-17-5p in *M. tuberculosis*-infected macrophages are verified to be Mcl-1 and STAT3 (a transcriptional activator of Mcl-1). Forced expression of miR-17-5p suppresses the interaction between Mcl-1 and beclin 1, resulting in the promotion of autophagy and acceleration of microbe killing. Furthermore, phosphorylation of protein kinase C (PKC) δ accelerates autophagy, and this effect is diminished by miR-17-5p (90). In BCG-infected RAW264.7 cells, enhanced expression of miR-17-5p prevents the maturation of phagosomes through the downregulation of Unc-51-like autophagy-activating kinase 1 (ULK1), an initiator of autophagy, and autophagosome-related protein LC3I/II, thereby increasing BCG propagation (91). Moreover, by targeting ATG7 and ATG16L, upregulated miR-20a attenuates autophagy and favors pathogen growth in BCG-infected macrophages (92). On the contrary, in H37Ra-infected THP-1 macrophages, downregulation of miR-106a suppresses bacterial proliferation as a result of promoted autophagy *via* increased levels of ULK1, ATG7, and ATG16L, which are important factors in autophagy (93). A study showed that overexpression of miR-129-3p induced by BCG in RAW264.7 cells is capable of attenuating *M. tuberculosis* killing *via* autophagy inhibition by targeting Atg4b, an ATG that contributes to the autophagosome formation step in which LC3I is converted into LC3II (94, 110). Furthermore, upregulation of miR-142-3p significantly abates H37Ra-induced autophagy by negatively controlling the expression of ATG16L and ATG4c, leading to the promotion of intracellular survival of the pathogen (95). *M. tuberculosis* replication is supported by overexpression of miR-33 and its passenger strand miR-33*, which target critical effectors that participate in autophagy such as ATG5, ATG12, LC3B, and lysosome-associated membrane protein 1 (LAMP1). Moreover, transcriptional regulators, including Forkhead box transcription factor class O (FOXO3) (111) and transcription factor EB (TFEB) (112), which promote the expression of genes implicated in autophagy biogenesis, are simultaneously repressed, resulting in impaired lipid catabolism (96).

Targeting ATG3, an E2-ubiquitin-like-conjugating enzyme participating in LC3-lipidation and autophagosome formation, virulent *M. tuberculosis*-induced miR-155 contributes to autophagy subversion to maintain bacterial survival (97). Meanwhile, miR-155 and miR-31 limit IFN-γ-induced autophagy by posttranscriptionally downregulating Ppp2r5a (99). In contrast, combined with the 3′ UTR of Ras homolog enriched in the brain (Rheb), overexpression of miR-155 accelerates the maturation of phagosomes and enhances autophagy in macrophages to eliminate intracellular *M. tuberculosis* (98).

Inversely correlated with beclin 1, miR-30A is overexpressed in THP-1 cells and AMs from bronchoalveolar lavage, inhibiting autophagy and thus permitting the immune escape of *M. tuberculosis*, but is expressed at a lower level after anti-TB treatment, indicating that miR-30A is a potential target and biomarker for treatment (100). UV radiation resistance-associated gene (UVRAG) can induce autophagosome formation in conjunction with beclin 1 (113). A profound increase in miR-125a-3p in *M. tuberculosis*-infected macrophages decreases the UVRAG protein level, inhibiting autophagosome maturation and prolonging intracellular pathogen survival (101). Interacting with UVRAG and LAMP1, DNA damage-regulated autophagy modulator 2 (DRAM2), an initiator of autophagy, is downregulated by miR-144* overexpression in human monocytes and macrophages after *M. tuberculosis* invasion, blocking autophagosome formation and inhibiting subsequent antimicrobial responses (102, 114). In addition, taking vacuolar protein sorting 33A (VPS33A) as the target, upregulated miR-432-5p suppresses the fusion between autophagosomes and lysosomes, playing a potentially critical role in the occurrence of ATB (103). Similarly, in the duration of phagolysosome biogenesis, miR-30a-3p and miR-30a-5p levels are elevated by recombinant ESAT-6 inc-treated differentiated THP-1 cells, impeding IL-18-mediated fusion between phagosomes and lysosomes and augmenting the survival of internalized *M. tuberculosis* (115). Furthermore, the promotion of miR-542-3p attenuates autophagy during *M. tuberculosis* infection by downregulating VPS11, resulting in the pathogen persistence in macrophages, whereas upregulation of VPS11 can counteract this effect (104).

Abundant expression of miR-27a in peripheral blood mononuclear cells (PBMCs) from ATB patients and primary peritoneal macrophages from H37Rv-infected mice is observed to promote *M. tuberculosis* intracellular residence. The rationale behind this effect is explained by the targeting of calcium voltage-gated channel auxiliary subunit alpha2delta3 (CACNA2D3), a transporter of Ca^2+^ located in the endoplasmic reticulum (ER), by miR-27a, which inhibits autophagy initiation (105). In addition, TNF-like weak inducer of apoptosis (TWEAK) is a target of miR-889, and an increased miR-889 level in latent tuberculosis infection (LTBI) individuals is related to autophagy suppression and *M. tuberculosis* maintenance in granulomas (106). Moreover, a team discovered that miR-25 is upregulated in *M. tuberculosis*- or BCG-infected macrophages, resulting in autophagy impairment and prolonged pathogen survival by blunting the function of the NPC1 protein, a cholesterol transporter located on the lysosomal membrane and involved in autophagolysosome formation (107). Furthermore, upregulation of miR-23a-5p in RAW264.7 cells and bone marrow-derived macrophages (BMDMs) prevents the induction of autophagy by targeting TLR2, thus conferring persistent *M. tuberculosis* existence in macrophages (108).

As previously stated, weakened autophagy resulting from *M. tuberculosis* infection is a considerable obstacle to clearing the microorganisms *via* the acid hydrolases deposited in the lysosomes, and may enable long-term bacterial survival inside the body, placing a heavy burden on the host immune system. Thus, the abovementioned studies on miRNAs may suggest promising therapeutic targets to counter autophagy subversion and opportunities to eliminate the pathogens.

### miRNA-regulated apoptosis in *M. tuberculosis* infection

**Figure 4:**
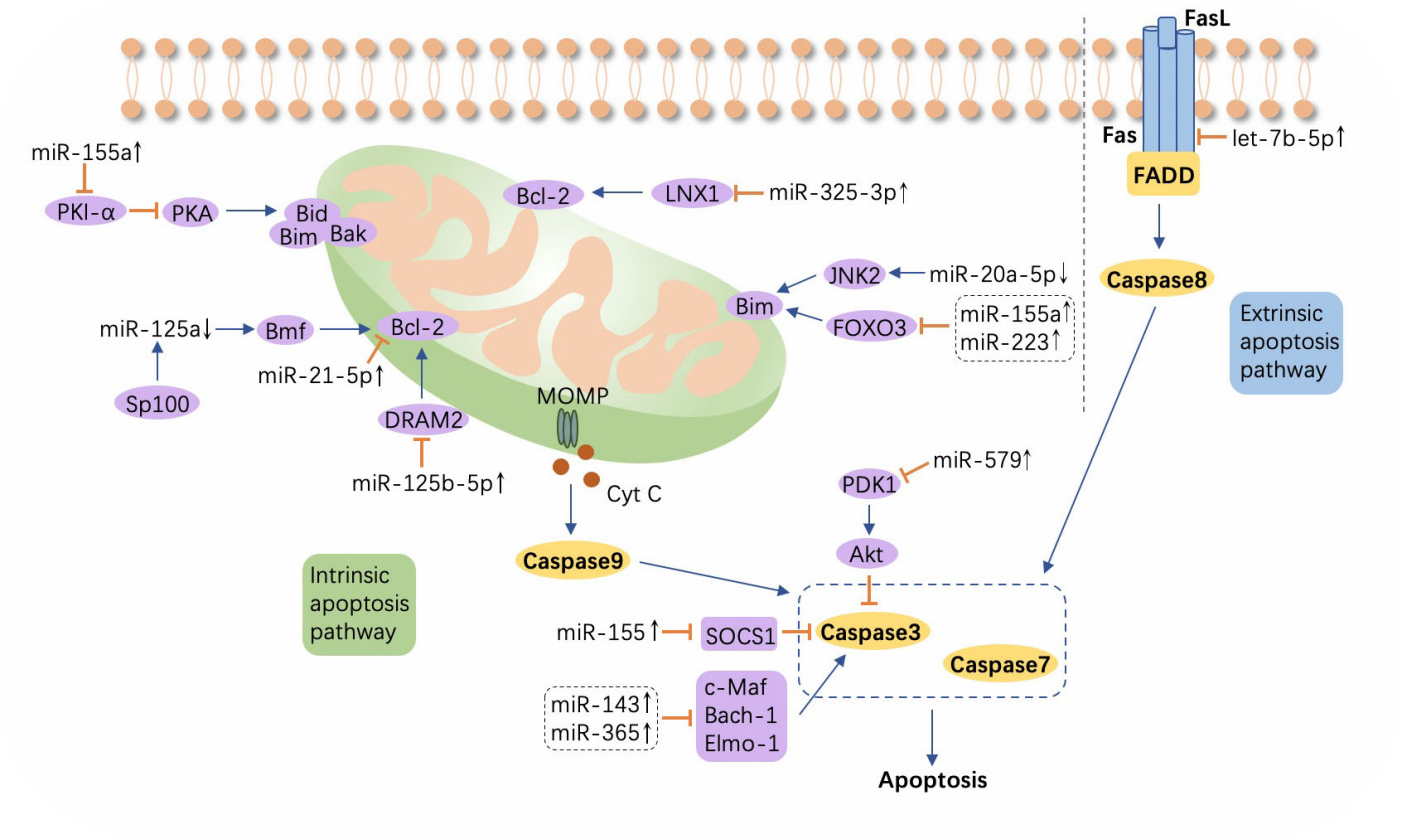
A schematic showing apoptosis and relevant microRNAs during *Mycobacterium tuberculosis* infection. Extrinsic apoptosis is activated by the combination of Fas and FasL, with the engagement of Fas-associated protein with DD (FADD) and the subsequent activation of caspase 8. In intrinsic apoptosis, cytochrome c (Cyt *c*) is released into the cytosol from mitochondria through mitochondrial outer membrane permeability (MOMP), which process is regulated by the Bcl-2 family. Altered microRNAs (miRNAs) target the Bcl-2 family or other molecules such as ligand of numb-protein X1 (LNX1), Forkhead box transcription factor class O (FOXO), DNA damage regulated autophagy modulator 2 (DRAM2), suppressor of cytokine signaling (SOCS), c-Maf, BTB and CNC homology 1 (Bach-1), and Engulfment and cell motility protein 1 (Elmo-1). ↑, upregulated; ↓, downregulated; ⟶, stimulate; ⟞, inhibit.

Apoptosis, a programmed cell demise mechanism that is triggered by an internal or external cellular stimulus, follows two patterns, intrinsic and extrinsic apoptosis, with both culminating in the activation of cysteine-aspartic proteases (caspases), enabling cell structure degradation and death. Cytochrome *c* (Cyt *c*), released into the cytosol from mitochondria through mitochondrial outer membrane permeability (MOMP), induces downstream caspase 9 to activate caspases 3 and 7, precursors in cell death *via* the intrinsic apoptosis pathway and the extrinsic pathway is initiated by the binding of Fas and FasL (116), which activates caspase 8. Bcl-2 family proteins have been identified as key modulators of Cyt *c* release into the cytoplasm during apoptosis and comprise anti-apoptotic (Bcl-2, Mcl-1, Bcl-XL, Bcl-W, etc.) and proapoptotic (Bim, Bid, Bax, Bak, PUMA, etc.) members (117, 118). Once the war between the host and *M. tuberculosis* begins, macrophages play a dual role by not only killing the pathogen *via* apoptosis but also by providing a natural niche for the invading microbe (119, 120). Moreover, the cunning bacteria can induce abnormal expression of host miRNAs to modulate Bcl-2 family activity, thereby either enhancing or inhibiting apoptosis (121) (**Figure 4**; **Supplementary Table S2**).

The expression of Jun N-terminal kinase 2 (JNK2), an upstream signaling gene that induces Bim expression, is observed to concomitantly increase with the downregulation of miR-20a-5p in *M. tuberculosis*-infected macrophages, resulting in the promotion of apoptosis and a higher rate of bacteria clearance (122). An increase in miR-21-5p, which targets Bcl-2 and TLR4, accelerates the apoptosis of macrophages and suppresses the secretion of inflammatory cytokines including IL-1β, IL-6, and TNF-α, which subsequently enhances *M. tuberculosis* survival (63). Overexpression of miR-125b-5p is associated with DRAM2 downregulation in H37Rv-infected macrophages, and an increase in apoptosis and a reduction in inflammation can protect macrophages when miR-125b-5p is forced into silencing, resulting in accelerated *M. tuberculosis* killing and suggesting a novel molecular therapeutic strategy for TB (123). Sp100, a nuclear body protein, suppresses miR-125a to increase the positive regulator of apoptosis, the Bcl2 modifying factor (Bmf), which promotes the elimination of the pathogen (124). Furthermore, when *Mycobacterium bovis* BCG attacks, upregulation of miR-155 targets protein kinase inhibitor-α (PKI-α) and activates the protein kinase A (PKA) signaling pathway to enhance proapoptotic genes, positively mediating macrophage apoptosis (125). ESAT6 can also induce overexpression of miR-155 in a TLR2-dependent manner, which inhibits SOCS1 expression while increasing caspase 3 activity and promoting macrophage apoptosis (126). Both of the aforementioned studies have verified apoptosis promotion and *M. tuberculosis* clearance facilitation induced by miR-155. However, miR-155 may act as a double-edged sword in the immune modulation, as monocyte apoptosis is inhibited by miR-155 *via* the suppressed expression of FOXO3, one of the transcription factors that induce apoptosis, leading to the subsistence of *M. tuberculosis* in PBMCs from ATB patients (127, 128).

In addition, elevated hsa-let-7b-5p and miR-223 inhibit apoptosis and enable enhanced *M. tuberculosis* survival in macrophages by downregulating Fas and FOXO3, respectively (129, 130). Similarly, in ATB patients, miR-582-5p is found to be upregulated and to suppress monocyte apoptosis by inhibiting FOXO1, attenuating bacteria elimination (131). miR-20b-5p carried in exosomes derived from *M. tuberculosis*-infected macrophages presents at a low level, inhibiting apoptosis by upregulating Mcl-1 and leading to acceleration of *M. tuberculosis* proliferation (132). The ligand of numb-protein X 1 (LNX1), an E3 ubiquitin ligase of NIMA-related expressed kinase 6 (NEK6), is directly targeted by miR-325-5p, which inhibits macrophage apoptosis through the activation of STAT3, a signaling pathway triggered by the accumulation of NEK6, ultimately leading to immune escape of *M. tuberculosis* (121). miR-143 and miR-365, highly overexpressed during *M. tuberculosis* infection, inhibit apoptosis by differentially downregulating c-Maf, Bach-1, and Engulfment and cell motility protein 1 (Elmo-1) in macrophages alternatively activated by IL-4/13. And the knockdown of these two miRNAs eases the *M. tuberculosis* burden and decreases chemokine CCL5 and IL-6 production (133). Moreover, ectopic overexpression of miR-579 in *M. tuberculosis*-infected macrophages intensifies cell apoptosis by negatively modulating phosphoinositide-dependent protein kinase 1 (PDK1) and sirtuin 1 (SIRT1), and this effect can be reversed by the accumulation of cPWWP2A, an endogenous sponge of miR-579 (134).

In addition to apoptosis and autophagy, necrosis, a biological process in the context of microbial invasion that is mediated by molecules such as cyclophilin-D (CypD) and p53, contributes to *M. tuberculosis*-mediated cytotoxicity in macrophages (119, 135). Virulent *M. tuberculosis* strains not only evade apoptosis but also induce necrotic cell death (136). However, miR-1281 can prevent trauma to macrophages from undergoing bacteria-induced necrosis and apoptosis by downregulating CypD expression (137).

Moreover, macrophages are capable of acquiring one of two phenotypes, the M1 and M2 phenotypes. When macrophages are polarized to the M1 type, they produce NO and inflammatory cytokines like TNF-α and IL-6, which promote an antibacterial response, whereas alternative activation triggers M2 polarization, which facilitates the production of anti-inflammatory cytokines such as IL-10 and arginase (138). As a member of the Krüpple-like factor (KLF) family, KLF4 activates M2 polarization and inhibits M1 polarization (139). C/EBPβ also plays a pivotal role in driving M2 polarization (140). Nucleotide-binding oligomerization domain-like receptor pyrin domain-containing protein 3 (NLPR3), a pivotal modulator in the inflammatory process, triggers the innate immune response with activation of caspase 1 and secretion of IL-1β and IL-18 (141). In a TB mouse model, miR-20b, which directly binds to NLRP3, is suppressed. After a miR-20b mimic is injected into TB mice, macrophages polarize into the M2 type, alleviating inflammation *via* the suppression of the NLRP3/caspase 1/IL-1β pathway, which may reflect a novel molecule-based therapeutic strategy (142). miR-26a, which has been observed to be downregulated during *M. tuberculosis* infection, elicits the upregulation of KLF4 and C/EBPβ, leading to microbe survival by inducing M2 polarization and repressing *M. tuberculosis* trafficking to lysosomes (109).

Taken together, the abovementioned studies showed that *M. tuberculosis*-induced alteration in the expression of host miRNAs facilitates the virulence of the bacteria and hinders their elimination. These effects are realized through diverse molecular mechanisms including regulation of signaling pathways, cytokine production, autophagy, and apoptosis. Therefore, pathogenesis of TB is likely based on various miRNAs, which may open up new exploratory avenues for developing innovative immunological therapies to eradicate this fierce bacterial foe.

## The role of lncRNAs in immune regulation in tuberculosis

Constituting a class of transcripts longer than 200 nucleotides, lncRNAs, the structures of which do not demonstrate high interspecies conservation but follow a tissue-specific expression pattern, directly modulate cellular processes but not by encoding proteins (10, 143, 144). In the human transcriptome, lncRNAs can be divided into several categories, such as intragenic and intergenic lncRNAs (145). It has been proven that lncRNAs play crucial regulatory roles in diverse biological processes, including cancer metastasis, through multiple mechanisms. For instance, certain lncRNA can sponge a miRNA by competing for binding sites in the miRNA, resulting in corresponding miRNA availability alterations and reducing its regulatory effect on specific mRNAs. Other mechanisms of lncRNA function in gene regulation, including modulating chromatin interactions and affecting gene expression *via* regulatory complex recruitment, have also been proposed (146–148). In terms of immunology, emerging evidence has confirmed the character of lncRNAs in modulating the mammalian immune response in host–pathogen interactions (149). For *M. tuberculosis* infection, we concluded relevant lncRNAs which are implicated in innate immune regulation and elaborated their potential roles in the pathogenetic process herein (**Table 3**).

**Table 3:** Long noncoding RNAs-mediated immune regulation in tuberculosis.

lncRNA	Expression	Targets	Biological function	Ref.
lincRNA-Cox2	↑	NF-κB, STAT3	Increases the production of TNF-α, IFN-γ, IL-6, Cox2, and iNOS, and inhibits apoptosis	(150, 151)
lncRNA PCED1B-AS1	↓	miR-155	Inhibits apoptosis and enhances autophagy	(152)
lnc-AC145676.2.1-6	↓	miR-29a	Inhibits CXCL10 secretion	(153)
lnc-TGS1-1	↓	miR-143	Inhibits apoptosis	(153)
lincRNA-EPS	↓	JNK/MAPK pathway	Inhibits apoptosis and enhances autophagy	(154)
lncRNA HOTAIR	↓	EZH2	Promotes transcription of DUSP4 and SATB1, and benefits *M. tuberculosis* survival	(155)
lncRNA-CD244	↑	EZH2	Inhibits the production of TNF-α, IFN-γ	(156)
lncRNA XLOC_012582	↑	SOCS3	Inhibits cytokine production	(157)
lncRNA XLOC_014219	↑	HMOX1	Affects the function of CD8^+^ T cells	(158)
LINC00870	↑	p-STAT5 and p-JAK2	Activates JAK/STAT signaling pathway and regulates cytokine production	(159)
lncRNA MIAT	↑	miR-665	Facilitates *M. tuberculosis* elimination	(160)
lnc-EST12	↓	FUBP3	Promotes NLRP3 inflammasome and GSDMD pyroptosis-IL-1β immune pathway	(161)
lncRNA XIST	↓	miR-125b-5p	Drives macrophages to M1 polarization	(162)

lncRNA, long noncoding RNA; Ref, reference; NF-κB, nuclear factor-κB; STAT, signal transducer and activator of transcription; TNF-α, tumor necrosis factor-α; IFN-γ, interferon-γ; IL, interleukin; Cox, cyclooxygenase; iNOS, inducible nitric oxide synthase; JNK/MAPK, Jun N-terminal kinase/mitogen-activated protein kinase; EZH2, enhancer of zeste homolog 2; DUSP4, dual specificity MAP kinase phosphatase 4; SATB1, special AT-rich sequence binding protein 1; *M. tuberculosis, Mycobacterium tuberculosis*; SOCS, suppressor of cytokine signaling; HMOX1, heme oxygenase 1; JAK, Janus kinase; FUBP3, far upstream element-binding protein; NLRP3, nucleotide-binding oligomerization domain-like receptor pyrin domain-containing protein 3; GSDMD, gasdermin D. ↑, upregulated; ↓, downregulated.

Induced by the *M. tuberculosis* H37Ra strain in macrophages, long intergenic noncoding RNA (lincRNA)-Cox2 is revealed to regulate inflammatory reactions in a broad-acting manner, with the promotion of TNF-α, IFN-γ, IL-6, Cox2, and iNOS production (150). Meanwhile, the knockdown of lincRNA-Cox2 inhibits the inflammatory response, promotes the apoptotic rate of H37Ra-infected macrophages, and facilitates pathogen proliferation *via* the suppression of the NF-κB and STAT3 signaling pathways (151). lncRNA PCED1B-AS1, which is downregulated in CD14^+^ monocytes from ATB patients, attenuates apoptosis and enhances autophagy by acting as an endogenous sponge of miR-155, leading to impeded *M. tuberculosis* elimination. FOXO3 and Rheb, genes targeted by miR-155, can reverse the impact of PCED1B-AS1 (152). miR-29a harbors a binding site for certain lncRNAs and has been verified to repress the secretion of CXCL10, negatively affecting T-cell recruitment after *M. tuberculosis* infection (163). Compared with the levels in healthy control (HC) groups, lnc-AC145676.2.1-6 and lnc-TGS1-1 are significantly decreased in TB patients, suppressing immune function *via* the elevation of miR-29a and miR-143, respectively, the functions of which have been enumerated above. Furthermore, the downregulation of lnc-TGS-1 is related to thrombocytopenia during TB treatment (153).

Downregulation of lincRNA-erythroid prosurvival (lincRNA-EPS) enhances autophagy by promoting LC3 and suppresses apoptosis, facilitating the eradication of the pathogen in BCG-infected RAW264.7 macrophages *via* the activation of JNK/mitogen-activated protein kinase (MAPK) signaling, which plays a key role in controlling the balance between apoptosis and autophagy (154). The expression of lncRNA HOX transcript antisense RNA (HOTAIR) has been found to be completely opposite in different *M. tuberculosis* strain-infected macrophages; it is upregulated in H37Ra-resided cells and suppressed in H37Rv-infected cells. Downregulation of lncRNA HOTAIR facilitates intracellular *M. tuberculosis* persistence due to the increased transcription of dual specificity MAP kinase phosphatase 4 (DUSP4) and special AT-rich sequence binding protein 1 (SATB1) by targeting enhancer of zeste homolog (EZH) 2 (155). Likewise, taking EZH2 as the target, lncRNA-CD244, which is induced by the T-cell inhibitory molecule CD244, suppresses the production of IFN-γ and TNF-α in *M. tuberculosis*-infected CD8^+^ T cells and attenuates the protective immunity of T cells to combat the invasion of the microorganisms (156).

The differential expression of 844 lncRNAs in B cells between individuals with or without TB was reported by Fu and his colleagues. Among these deregulated lncRNAs, lncRNA XLOC_012582 is highly expressed in B cells from TB patients, along with SOCS3 promotion, an inhibitor of cytokine secretion. However, the relationship between the altered expression of XLOC_012582 and SOCS3 and the particular impact of this change on *M. tuberculosis* infection remains to be further explored (157). Meanwhile, the team revealed the upregulation of lncRNA XLOC_014219 and a decrease in heme oxygenase 1 (HMOX1) in CD8^+^ T cells from ATB individuals. Nevertheless, whether this phenomenon is involved in the dysfunction of CD8^+^ T cells has not been clarified (158). LINC00870, which is significantly induced by *M. tuberculosis* in PBMCs, mediates the immune response against the bacteria by suppressing the ability of Th1 cells to produce cytokines such as IL-2 and IFN-γ and promoting the expression of cytokines generated by Th2 cells including IL-4 and IL-10. These impacts may be attributable to the activation of the Janus kinase (JAK)/STAT signaling pathway *via* the acceleration of the expression of p-STAT5 and p-JAK2 mediated by LINC00870 (159). Another study illuminated that the expression level of lncRNA MIAT is heightened, which accelerates autophagy and apoptosis in BCG-infected THP-1 macrophages by binding to miR-665 to enhance ULK1 activation, ultimately inhibiting intracellular pathogen maintenance (160).

Inhibited by the *M. tuberculosis* Rv1579c *via* the activation of the JAK2-STAT5a pathway, lnc-EST12 decreases the production of IL-1β, IL-6, and CCL5/8. Moreover, it suppresses the NLRP3 and gasdermin D (GSDMD) pyroptosis-IL-1β pathway by binding too far upstream element-binding protein (FUBP) 3 to suppress innate immunity toward *M. tuberculosis* (161). Negative pressure therapy, promoting wound healing *via* vacuum dressings, has been utilized as a TB treatment and has shown promising effects. Upregulation of lncRNA XIST and downregulation of miR-125b-5p during the infection can be reversed by this treatment strategy. Through modulation of the lncRNA XIST/miR-125b-5p/A20/NF-κB axis, which ultimately increases the activity of NF-κB p65, this regimen facilitates the polarization of macrophages to the M1 phenotype, enhancing the inflammatory response to reduce *M. tuberculosis* survival (162).

As discussed above, lncRNAs regulate the innate immune response to *M. tuberculosis* in various ways, including serving as sponges of miRNAs and regulating signaling pathways, which implies that we should undertake related research for the exploration of novel therapies. However, existing studies are not sufficiently systematic for organized analysis, and certain results remain unverified. Therefore, more investigations are yet to be done.

## The role of circRNAs in immune regulation in tuberculosis

Due to the noncanonical splicing process, back-splicing, the structure of circRNAs is thoroughly distinct from that of other RNA molecules. They are covalently closed, without typical components such as 5′ capping and 3′ polyadenylation (164). Viroids were the first circRNAs to be discovered (165). With considerable progress in high-throughput RNA sequencing technology and bioinformatics, thousands of circRNAs have been identified in eukaryotes (166), demonstrating a tissue- and cell-specific expression pattern (167, 168). Acting as miRNA sponges, the more binding sites circRNAs contain, the more competitive rivalry there will be (169, 170). Furthermore, circRNAs can enhance the function of certain proteins by interacting with RNA-binding proteins (RBPs) (171) and serve as scaffolds in various processes involving enzymes and their substrates (172). Continuous evidence has confirmed that circRNAs exert a nonnegligible influence on numerous cellular processes, from cell cycle control to cancer development (173, 174).

With respect to infection, previous studies have found that circRNAs perform flexible roles when viruses invade (175). Several studies on TB have been carried out and have revealed the impact of circRNAs on the modulation of the host immune response (**Supplementary Table S3**). For example, circAGFG1, which is upregulated in TB patients, promotes autophagy and inhibits apoptosis by targeting miR-1257 in macrophages, which subsequently increases the Notch 2 level (176). Another study found that circTRAPPC6B is downregulated during *M. tuberculosis* infection. Furthermore, forced expression of this circRNA antagonizes the capacity of miR-874-3p to inhibit autophagy by targeting ATG16L, thereby increasing autophagy sequestration and restricting intracellular pathogen growth (177). Moreover, circRNA-0003528 enhances *M. tuberculosis*-related macrophage polarization, which is mediated by the promotion of CTLA4 *via* the inhibition of miR-224-5p, miR-324-5p, and miR-488-5p (178). Deng et al. discovered that circ_0001490 suppresses *M. tuberculosis* survival and promotes the viability of host macrophages by sponging miR-579-3p to increase the expression of FSTL1 (179). Similarly, hsa_circ_0045474 is downregulated and plays a positive role in autophagy induction by promoting the expression of miR-582-5p and suppressing the expression of the downstream target TNKS2 in *M. tuberculosis*-infected macrophages, facilitating the bacterial clearance in the end (180).

As demonstrated, the rarity and unconfirmed results of relevant studies lead to a vague understanding of circRNAs’ function in TB. Because circRNAs present peculiar structures, a cyclic construction formed through covalent bonds, they are highly stable and resistant to degradation mediated by exonucleases (164). Therefore, circRNAs may be ideal biomarkers for TB detection and prognosis prediction due to their aberrant expression (181), and this possibility is discussed, along with other potential ncRNA biomarkers, in the following section.

## Noncoding RNAs as biomarkers in tuberculosis

On account of the huge burden of the disease, accurate diagnosis of TB is important but remains a formidable challenge. Conventional methods based on sputum microscopy or culture are time- consuming and resource-limited. Moreover, there is a grim fact that the incidence of DR-TB continues to increase, leading to lower treatment effectiveness (2). In addition, adverse drug reactions (ADRs) to anti-TB chemotherapy complicate disease management (182, 183). Thus, novel, precise, and efficient indicators for diagnosis, drug resistance prediction, and treatment monitoring of TB are urgently required. Based on differentially expressed ncRNAs, related studies have identified numerous biomarkers with various functions as follows.

### Diagnostic biomarkers of pulmonary tuberculosis

As mentioned above, miR-432-5p, upregulated during *M. tuberculosis* infection, inhibits the fusion between autophagosomes and lysosomes by targeting VPS33A. In the meantime, the expression of miR-17-5p and miR-20b-5p is significantly elevated in the serum of TB patients. Therefore, one group developed a diagnostic model for TB *via* the combination of the three miRNAs, achieving an area under the curve (AUC) of 0.908 (103). As previously mentioned, miR-889 inhibits autophagy by targeting TWEAK and can serve as a biomarker for LBTI and a potential therapeutic target due to its high expression in patients, which is decreased after prophylactic therapy (106). Another study noticed that miR-29a and miR-99b are upregulated while miR-21, miR-146a, and miR-652 are decreased in the plasma of TB patients. Therefore, the team constructed a model based on these five miRNAs to determine *M. tuberculosis* infection, showing an AUC of 0.976 (184). After obtaining this result, they utilized a combination of proteins and miRNAs to further improve the model (185). Another group combined miR-142-3p with electronic health record (EHR) data to detect TB, and the AUC reached 0.94 (186). Compared with LTBI patients, hsa-miR-1246, hsa-miR-2110, hsa-miR-370-3p, hsa-miR-193b-5p, and hsa-miR-28-3p are specifically expressed in TB cohorts (187). In addition, miR-185-5p is more highly expressed in plasma exosomes obtained from TB patients than in HCs, demonstrating a diagnostic potential with an AUC of 0.75 (21).

Regarding lncRNAs, considerable effort has also been directed to explore their capacity for TB detection. Consisting of four lncRNAs including NR_038221, NR_003142, ENST00000570366, and ENST00000422183, the AUC of a diagnostic model was reported to be 0.845 (188). Hu and his team identified that ENST00000497872, n333737, and n335265 are differentially expressed in clinically diagnosed TB patients and established a nomogram to predict the infection by taking advantage of the data on these three lncRNAs and six clinical covariates such as age and hemoglobin, with an AUC equal to 0.89 (189). Similarly, lncRNAs TCONS_00001838 and n406498 are significantly differentially expressed in TB patients. Combining the two lncRNA loci with eight EHR indicators through logistic regression, the model achieved a decent predictive value of TB with an AUC of 0.86 (190). Another study discovered that lncRNA CCAT1 is upregulated in TB patients, concomitant with high mortality rates, and is negatively correlated with IL-10 (191). Furthermore, the level of LINC00870 is higher in the plasma and sputum obtained from TB and LTBI patients and decreases after 3 months of anti-TB therapy (ATT), showing the potential of being a biomarker for the diagnosis and treatment evaluation of TB (159).

In terms of circRNAs, the significant dysregulation of hsa_circ_0043497 and hsa_circ_0001204 in PBMCs from TB patients indicated their diagnostic capacity, as their AUCs achieved at 0.860 and 0.848, respectively (192). Another group validated the overexpression of circRNA_051239, circRNA_029965, and circRNA_404022 in the serum, and constructed a panel of the three circRNAs for use in TB diagnosis, reaching an AUC of 0.992 (193). Moreover, hsa_circRNA_001937 is significantly increased in PBMCs from TB patients compared to patients with lung cancer, pneumonia, or chronic obstructive pulmonary disease, demonstrating the potential of this circRNA to be a diagnostic biomarker with an AUC of 0.873, and it is deemed a candidate molecule for measuring TB severity (194).

As mentioned above, utilizing a combination of various ncRNAs, several diagnostic models have shown favorable performance, inspiring us to develop creative approaches based on these molecules to detect TB. Rather than waiting for the test results of pathogens, physicians can rapidly determine *M. tuberculosis* infection through fast evaluation of blood or other conveniently obtained clinical samples (**Table 4**).

**Table 4:** Noncoding RNAs as biomarkers for tuberculosis diagnosis.

ncRNAs	Study sample	Expression	Performance	Ref.
miR-423-5p, miR-17-5p, miR-20b-5p	Serum	↑	AUC 0.908	(103)
miR-889	Plasma	↑	NA	(106)
miR-29a, miR-99b	Plasma	↑	AUC 0.976	(184)
miR-21, miR-146a, miR-652	Plasma	↓	AUC 0.976	(184)
miR-142-3p	Serum	↓	AUC 0.94	(186)
hsa-miR-1246, hsa-miR-2110, hsa-miR-370-3p, hsa-miR-28-3p, hsa-miR-193b-5p	Exosome	↑	NA	(187)
miR-185-5p	Exosome	↑	AUC 0.75	(21)
lncRNAs NR_038221, NR_003142, ENST00000570366	Plasma	↑	AUC 0.845	(188)
lncRNA ENST00000422183	Plasma	↓	AUC 0.845	(188)
lncRNAs ENST00000497872, n333737	PBMCs	↓	AUC 0.89	(189)
lncRNA n335265	PBMCs	↑	AUC 0.89	(189)
lncRNA TCONS_00001838	PBMCs	↑	AUC 0.86	(190)
lncRNA n406498	PBMCs	↓	AUC 0.86	(190)
lncRNA CCAT1	Plasma	↑	NA	(191)
LINC00870	Sputum and plasma	↑	NA	(159)
hsa_circ_0043497	PBMCs	↑	AUC 0.860	(192)
hsa_circ_0001204	PBMCs	↓	AUC 0.848	(192)
circRNA_051239, circRNA_029965, circRNA_404022	Serum	↑	AUC 0.992	(193)
hsa_circRNA_001937	PBMCs	↑	AUC 0.873	(194)

ncRNA, noncoding RNA; Ref, reference; AUC, area under the curve; NA, not available; PBMCs, peripheral blood mononuclear cells. ↑, upregulated; ↓, downregulated.

### Biomarkers for early detection of active tuberculosis

A study discovered that 24 miRNAs are upregulated and 6 miRNAs are downregulated in ATB patients, among which the significantly overexpressed hsa-miR-196b and hsa-miR-376c demonstrate the greatest potential to be ATB indicators (195). Presenting a higher fold change in ATB patients than in HCs, miR-155* and miR-155 can also serve as diagnostic biomarkers for ATB, with respective AUC of 0.7945 and 0.8972 (196). Moreover, a nested case-control study revealed that, combined with body mass index (BMI) and TB history, downregulated hsa-miR-16-5p and hsa-miR-451a can contribute to the prediction of developing ATB from LBTI, with AUCs of 0.84 and 0.85, respectively (197). Shown to be increased in the serum of ATB patients, miR-96, miR-425, and miR-484 are also diagnostic candidates, showing moderate performance with AUC values ranging from 0.62 to 0.72 (198).

Notably, after synthesizing analysis of the NONCODE database and GEO dataset, Fang and his colleagues discovered the ability of four lncRNAs, NONHSAT101518.2, NONHSAT067134.2, NONHSAT148822.1, and NONHSAT078957.2, to discriminate ATB patients from HCs *via* plasma samples, with AUCs ranging from 0.7080 to 0.9502 (199).

In addition, overexpressed circRNA_103017, circRNA_059914, and circRNA_101128 are confirmed to be increased by *M. tuberculosis*, while circRNA_062400 is downregulated in PBMCs from ATB individuals. Among these circRNAs, circRNA_103017 shows the maximum potential as an indicator for ATB diagnosis, with an AUC of 0.870 (200). Furthermore, the upregulation of hsa_circ_002883 in PBMCs endows it with the power to be a candidate biomarker for ATB determination, showing significant discrimination efficiency with an AUC of 0.773 (201). In contrast, hsa_circRNA_103571 expression is obviously decreased in plasma from ATB patients, with a diagnostic ability for identifying ATB (AUC 0.838) (202). Moreover, the lower expression of hsa_circ_0005836 in PBMCs from the ATB cohort is verified, indicating that it may serve as a novel biomarker for *M. tuberculosis* infection (22) (**Supplementary Table S4**).

To control the spread of TB, proactively recognizing and ameliorating active individuals is of imperative priority. Although the abovementioned studies demonstrate decent performance, the clinical practicality of using these molecules for diagnosis remains to be verified in realistic medical settings. After being strictly tested, these methods may be novel diagnostic decision-making tools, providing opportunities to carry out proper treatment against ATB as early as possible.

### Biomarkers for prediction of tuberculosis drug resistance

Considering the roles of ncRNAs in identifying TB drug resistance, a few studies have also been carried out to analyze their potential in this daunting task. For example, in DR-TB patients, miR-197-3p and miR-223-3p are downregulated, while miR-let-7e-5p is increased, and a multivariate analysis based on these three miRNAs was performed to distinguish resistant individuals from HCs. Finally, the diagnostic model achieved an AUC of 0.96 for DR-TB recognition. A favorable outcome showing an AUC of 0.95 was observed for multidrug-resistant tuberculosis (MDR-TB) subgroup identification (203). Moreover, the expression of miR-378 in the serum is associated with adverse therapeutic consequences since it presented a higher level in MDR-TB than in single-drug-resistant tuberculosis (SDR-TB). With an AUC of 0.767, this miRNA can also distinguish the ATB population from the LBTI patients (204). Furthermore, lncRNA n335659 shows a statistically significant downward trend in the MDR-TB group compared with the HC cohort; hence, it may act as a latent biomarker for TB drug resistance prediction as well (205) (**Supplementary Table S5**).

Nonetheless, predicting drug resistance in TB through differentially expressed ncRNAs is still a challenging task, as there are not abundant or sufficiently comprehensive studies that have been validated in actual clinical environments. Therefore, we need to continue to explore the hidden potential of ncRNAs in DR-TB recognition to realize early diagnosis of drug resistance and carry out effective treatment.

### Biomarkers for treatment evaluation of tuberculosis

During anti-TB treatment, drug-induced liver injury (DILI) is the most common severe adverse drug response, with an approximate incidence of 2%–28% (206). miR-122 and miR-192 are significantly decreased in the serum of TB patients with DILI and could be used to predict this serious complication in TB (207). Likewise, another study revealed the value of upregulated circMARS in identifying TB patients suffering from anti-TB drug-induced liver injury (ADLI), with an AUC of 0.80 (208). Moreover, the lower expression of lnc-TGS1-1 is capable of indicating the development of thrombocytopenia after treatment (153).

Used for treatment response monitoring, the expression of hsa-miR-346 is significantly increased in the supernatant of macrophages and serum during *M. tuberculosis* infection but declines after two months of ATT (209). Moreover, the upregulation of miR-29a and miR-99b in patients with TB is attenuated, reaching levels equal to those in HCs after the completion of treatment (184). Similarly, LINC00870 is overexpressed in both sputum and plasma samples from TB or LBTI patients, but its expression is reduced after three months of ATT (159). In addition, the expression of lncRNA CCAT1 also decreases during ATT, and a higher level of CCAT1 is correlated with high mortality (191), as briefly described in **Supplementary Table S5**.

The treatment monitoring capacity of ncRNAs in response evaluation and side effect prediction of anti-TB chemotherapy has provided researchers with a promising prospect to precisely assess the curative effect in patients with TB and to adjust therapy regimens on an individual basis.

## Conclusion remarks

In summary, we concluded the latest released original studies investigating the promising roles of ncRNAs in *M. tuberculosis* infection, elucidating their functions, which vary in pathogenesis and as biomarkers. The research referenced may shed light on the principles behind novel TB therapeutic schemes based on those seemingly negligible but indeed crucial elements. Moreover, owing to the differential expression of ncRNAs in the clinical samples of patients with different statuses, a new chapter is added to the diagnosis, drug resistance prediction, and treatment monitoring of TB, with the further purpose of making appropriate clinical decisions.

Despite the attainment obtained in the field of ncRNAs in TB, pitfalls remain in the path of integrating these critical molecules into a realistic clinical scenario. First, since previous studies have linked various ncRNAs to the pathogenesis of TB, which may be instructive for HDT development, novel remedies for TB based on these ncRNAs are promising to explore, but relevant investigations are lacking, making their use in treatment infeasible in practice. Second, a single ncRNA can interfere with the expression of diverse genes, but the complete targets of ncRNAs have not been explored thoroughly yet (210). Thus, it is rational that one ncRNA may lead to paradoxical effects in *M. tuberculosis* infection, making determining a therapeutic strategy complicated and confusing. Third, although the parameters of the diagnostic models based on ncRNAs seem to be promising with decent AUCs, rigorous and prospective trials of these ncRNAs tested in substantial samples are required to verify their true potential as biomarkers in TB. Another considerable issue is that a large proportion of the studies to date have focused on miRNAs, with research advancements made with lncRNAs, circRNAs, and other ncRNAs being relatively rare. Prominent advances in RNA sequencing have opened the gate to an extensive domain from which to determine their function, and related work in the future is warranted.

In conclusion, the research field of ncRNAs in *M. tuberculosis* infection has met a certain degree of success in pathogenesis and biomarker exploration and holds broad promise. However, several challenges still need to be addressed before these molecules can be seamlessly integrated into clinical practice to enrich personalized and creative diagnostic strategies (211, 212), and further direct treatment decision-making in TB, especially for the management of drug-resistant patients.

## Author contributions

WL and CW designed the conception of the manuscript. SL, JM, JS, JL and YZ drafted the original version of the manuscript and drew the figures and tables. ZW, HG and CW revised the final version of the manuscript. All authors contributed to the article and approved the submitted version.

## Funding

The study was supported by the National Natural Science Foundation of China (82100119, 92159302), the Science and Technology Project of Sichuan (2022ZDZX0018, 2020YFG0473), the Chinese Postdoctoral Science Foundation (2022T150451, 2021M692309), and the Postdoctoral Program of Sichuan University (2021SCU12018).

## Conflict of interest

The authors declare that the research was conducted in the absence of any commercial or financial relationships that could be construed as a potential conflict of interest.

## Publisher’s note

All claims expressed in this article are solely those of the authors and do not necessarily represent those of their affiliated organizations, or those of the publisher, the editors and the reviewers. Any product that may be evaluated in this article, or claim that may be made by its manufacturer, is not guaranteed or endorsed by the publisher.
