# Supplementary material for: Immune regulation and emerging roles of noncoding RNAs in Mycobacterium tuberculosis infection
Source: Front Immunol. 2022 Oct 13;13:987018. doi: 10.3389/fimmu.2022.987018 (PMC9608867; doi:10.3389/fimmu.2022.987018)
Supplement: Supplementary file 1 [file Table_1.docx]

Supplementary Materials

## Supplementary Tables

**Table S1. MicroRNA-regulated cytokine secretion in tuberculosis**

| **miRNAs** | **Expression** | **Targets** | **Biological function** | **Ref.** |
| --- | --- | --- | --- | --- |
| miR-29 |  | IFN-γ mRNA | Increases the secretion of IFN-γ | 67 |
| miR-132, miR-26a |  | p300 | Inhibit the secretion of IFN-γ | 70 |
| miR-144* |  | NA | Inhibits the secretion of TNF-α and IFN-γ | 71 |
| miR-125b |  | TNF-α mRNA | Inhibits the secretion of TNF-α | 72 |
| miR-155 |  | SHIP1 | Inhibits the secretion of TNF | 72 |
|  |  | SHIP1, Bach1 | Inhibits the secretion of IL-6 and Cox-2 | 73 |
|  |  | C/EBPβ | Decreases the synthesis of NO | 74 |
| miR-32-5p |  | FSTL1 | Inhibits the secretion of IL-1β, IL-6, and TNF-α | 79 |
| miR-378d |  | Rab10 | Increases the secretion of IL-1β, IL-6, and TNF-α | 80 |
| miR-21 |  | IL-12p35 | Suppresses the production of IL-12 | 81 |
| miR-206 |  | TIMP3 | Increases the secretion of IL-1β, IL-6, TNF-α, and IFN-γ | 82 |
| miR-222-3p |  | PTEN | Suppresses the production IL-6, IL-1β, and TNF-α | 83 |
| miR-495 |  | SOD2 | Inhibits the production of ROS | 84 |

miRNA, microRNA; Ref, reference; IFN-γ, interferon-γ; mRNA, messenger ribose nucleic acid; NA, not available; TNF-α, tumor necrosis factor-α; SHIP1, SH2-containing inositol 5’-phosphatase; Bach1, BTB and CNC homology 1; IL, interleukin; Cox, cyclooxygenase; C/EBPβ, CCAAT/enhancer binding protein β; NO, nitric oxide; FSTL1, Follistatin-like protein 1; TIMP3, tissue inhibitor of matrix metalloproteinase 3; PTEN, phosphatase and tensin; SOD2, superoxide dismutase 2; ROS, reactive oxygen species. , upregulated; , downregulated.

**Table S2. MicroRNA-regulated apoptosis in tuberculosis**

| **miRNAs** | **Expression** | **Targets** | **Biological function** | **Ref.** |
| --- | --- | --- | --- | --- |
| miR-20a-5p |  | JNK2 | Induces the expression of Bim and promotes apoptosis | 122 |
| miR-21-5p |  | Bcl2 | Represses apoptosis | 63 |
| miR-125b-5p |  | DRAM2 | Inhibits the expression of IL-10 and Bcl-2, and represses apoptosis | 123 |
| miR-125a |  | Bmf | Promotes apoptosis | 124 |
| miR-155 |  | PKI-α | Enhances PKA signaling pathway and promotes apoptosis | 125 |
|  |  | SOCS1 | Promotes apoptosis | 126 |
|  |  | FOXO3 | Inhibits apoptosis | 127 |
| hsa-let-7b-5p |  | Fas | Represses apoptosis and ameliorates *M. tuberculosis* survival | 129 |
| miR-223 |  | FOXO3 | Represses apoptosis of macrophages | 130 |
| miR-582-5p |  | FOXO1 | Represses apoptosis of monocytes | 131 |
| miR-20b-5p |  | Mcl-1 | Represses apoptosis body formation and vacuolization | 132 |
| miR-325-3p |  | LNX1 | Represses apoptosis by activation of STAT3 signaling pathway | 121 |
| miR-143,  miR-365 |  | c-Maf, Bach-1, Elmo-1 | Repress apoptosis and increase the production of CCL5 and IL-6 | 133 |
| miR-579 |  | SIRT, PDK1 | Enhances apoptosis and cytotoxicity of macrophages | 134 |

miRNA, microRNA; Ref, reference; JNK, Jun N-terminal kinase 2; DRAM2, DNA damage regulated autophagy modulator 2; IL, interleukin; Bmf, Bcl2 modifying factor; PKI-α, protein kinase inhibitor peptide-α; PKA, protein kinase A; SOCS, suppressor of cytokine signaling; FOXO, Forkhead box transcription factor class O; *M. tuberculosis*, *Mycobacterium tuberculosis*; LNX1, Ligand of numb-protein X 1; STAT, signal transducer and activator of transcription; Bach-1, BTB and CNC homology 1; Elmo-1, Engulfment and cell motility protein 1; CCL, chemokine (C–C motif) ligand; SIRT, sirtuin 1; PDK1, phosphoinositide-dependent protein kinase 1. , upregulated; , downregulated.

**Table S3. Circular RNA-mediated immune regulation in tuberculosis**

| **circRNA** | **Expression** | **Targets** | **Biological function** | **Ref.** |
| --- | --- | --- | --- | --- |
| circAGFG1 |  | miR-1257 | Promotes autophagy and inhibits apoptosis | 176 |
| circTRAPPC6B |  | miR-874-3p | Inhibits autophagy | 177 |
| circRNA-0003528 |  | miR-224-5p, miR-324-5p, miR-488-5p | Promotes *M. tuberculosis*-related macrophages polarization | 178 |
| circ_0001490 |  | miR-579-3p | Inhibits inflammatory response and promotes *M. tuberculosis* survival | 179 |
| hsa_circ_0045474 |  | miR-582-5p | Positively regulates autophagy induction | 180 |

circRNA, circular RNA; Ref, reference; *M. tuberculosis*, *Mycobacterium tuberculosis*. , upregulated; , downregulated.

**Table S4. Noncoding RNAs in active tuberculosis detection**

| **ncRNAs** | **Study Sample** | **Expression** | **Performance** | **Ref.** |
| --- | --- | --- | --- | --- |
| hsa-miR-196b, hsa-miR-376c | Serum |  | NA | 195 |
| miR-155, miR-155* | PBMCs |  | AUC 0.7945-0.8972 | 196 |
| hsa-miR-16-5p, hsa-miR-451a | Serum |  | AUC 0.84-0.85 | 197 |
| miR-96, miR-425, and miR-484 | Exosome |  | AUC 0.62-0.72 | 198 |
| miR-378 | Serum |  | AUC 0.767 | 204 |
| lnc-NONHSAT101518.2, NONHSAT067134.2, NONHSAT148822.1, NONHSAT078957.2 | Plasma |  | AUC 0.7080-0.9502 | 199 |
| circRNA_103017 | PBMCs |  | AUC 0.870 | 200 |
| hsa_circ_002883 | PBMCs |  | AUC 0.773 | 201 |
| hsa_circRNA_103571 | Plasma |  | AUC 0.838 | 202 |
| hsa_circ_0005836 | PBMCs |  | NA | 22 |

ncRNA, noncoding RNA; Ref, reference; PBMCs, peripheral blood mononuclear cells; NA, not available; AUC, are under the curve. , upregulated; , downregulated.

**Table S5. Drug resistance prediction, side effect, and treatment response estimation of noncoding RNAs in tuberculosis**

| **Function** | **ncRNAs** | **Study Sample** | **Expression** | **Performance** | **Ref.** |
| --- | --- | --- | --- | --- | --- |
| **Drug resistance prediction** | miR-197-3p, miR-223-3p | Serum |  | AUC 0.95-0.96 | 203 |
|  | miR-let-7e-5p | Serum |  | AUC 0.95-0.96 | 203 |
|  | miR-378 | Serum |  | NA | 204 |
|  | lncRNA n335659 | Serum |  | NA | 205 |
| **Side effect prediction** | miR-122 and miR-192 | Serum |  | NA | 207 |
|  | circMARS | Serum |  | AUC 0.80 | 208 |
|  | lnc-TGS1-1 | Whole blood |  | NA | 153 |
| **Treatment response estimation** | hsa-miR-346 | Supernatant of macrophages and serum |  | NA | 209 |
|  | miR-29a, miR-99b | Plasma |  | NA | 184 |
|  | LINC00870 | Sputum or plasma |  | NA | 159 |
|  | lncRNA CCAT1 | Plasma |  | NA | 191 |

ncRNA, noncoding RNA; Ref, reference; AUC, area under the curve; NA, not available. , upregulated; , downregulated.
